# An Atypical Presentation of Upper Motor Neuron Predominant Juvenile Amyotrophic Lateral Sclerosis Associated with *TARDBP* Gene: A Case Report and Review of the Literature

**DOI:** 10.3390/genes13081483

**Published:** 2022-08-19

**Authors:** Daniel Sánchez-Tejerina, Juan Luis Restrepo-Vera, Eulalia Rovira-Moreno, Marta Codina-Sola, Arnau Llauradó, Javier Sotoca, Maria Salvado, Núria Raguer, Elena García-Arumí, Raúl Juntas-Morales

**Affiliations:** 1 Neuromuscular Diseases Unit, European Reference Network on Rare Neuromuscular Diseases (ERN EURO-NMD), Department of Neurology, Vall d’Hebron University Hospital, Passeig de la Vall d’Hebron, 119, 08035 Barcelona, Spain; 2 Department of Clinical and Molecular Genetics, Vall d’Hebron University Hospital, Universitat Autònoma de Barcelona, 08035 Barcelona, Spain; 3 Department of Clinical Neurophysiology, Vall d’Hebron University Hospital, Passeig de la Vall d’Hebron, 119, 08035 Barcelona, Spain

**Keywords:** *TARDBP*, TDP-43, juvenile amyotrophic lateral sclerosis, upper motor neuron predominant disorder

## Abstract

Amyotrophic lateral sclerosis (ALS) is a neurodegenerative disease that can rarely affect young individuals. Juvenile ALS (JALS) is defined for individuals with an onset of the disease before the age of 25. The contribution of genetics to ALS pathology is a field of growing interest. One of the differences between adult-onset ALS and JALS is their genetic background, with a higher contribution of genetic causes in JALS. We report a patient with JALS and a pathogenic variant in the *TARDBP* gene (c.1035C > G; p.Asn345Lys), previously reported only in adult-onset ALS, and with an atypical phenotype of marked upper motor neuron predominance. In addition, the proband presented an additional variant in the *NEK1* gene, c.2961C > G (p.Phe987Leu), which is classified as a variant of unknown significance. Segregation studies showed a paternal origin of the *TARDBP* variant, while the variant in *NEK1* was inherited from the mother. We hypothesize that the *NEK1* variant acts as a disease modifier and suggests the possibility of a functional interaction between both genes in our case. This hypothesis could explain the peculiarities of the phenotype, penetrance, and the age of onset. This report highlights the heterogeneity of the phenotypic presentation of ALS associated with diverse pathogenic genetic variants.

## 1. Introduction

Amyotrophic lateral sclerosis (ALS), classically regarded as the most common motor neuron disease, is now recognized as a multisystemic neurodegenerative disorder with great heterogeneity in its clinical and pathophysiological aspects [1]. Research in the last decades has revealed multiple pathological pathways involved, including various genetic aspects and the description of several causative genes [2,3]. Although it predominantly affects adults, in a small percentage of cases, young individuals are affected. Juvenile ALS (JALS) is typically defined for individuals with an onset of the disease before the age of 25 [4,5].

One of the main differences between adult-onset ALS and JALS is their genetic background, with a higher contribution of genetic causes detected in JALS (40%) than in adult-onset ALS (around 10%) [2,6,7]. The frequencies of associated genes are also different between the two forms of onset. A recent review of JALS [7] identified *FUS* (fused in sarcoma), *SETX* (Senataxin), and *ALS2* (Alsin) as the most common genes reported in the literature. In contrast, pathogenic variants in genes commonly associated with adult forms, such as *SOD1* (copper–zinc superoxide dismutase) or *TARDBP* (transactive response DNA-binding protein), were reported less frequently, and *C9orf72* (chromosome 9 open reading frame 72), the most prevalent inherited gene in adult disease, has not been reported in childhood [7,8].

We report herein a patient with JALS and a pathogenic variant in the *TARDBP* gene (c.1035C > G, p.Asn345Lys), previously reported only in adult-onset ALS, and with an atypical phenotype of marked upper motor neuron predominance. In addition, we performed a literature review of JALS cases associated with variants in the *TARDBP* gene to discuss the phenotype.

## 2. Patient and Method

### 2.1. Clinical Presentation

A 24-year-old male patient of Spanish origin with no relevant medical history presented to our neuromuscular disease unit with a progressive disorder of rigidity and weakness of the lower limbs. The symptoms had started 3 months earlier, initially affecting the left lower limb and causing difficulty in walking. The first neurological examination revealed signs of upper motor neuron involvement with hyperreflexia and spasticity in all four limbs, a sustained left ankle clonus, bilateral Babinski sign, and bilateral Hoffman sign. There was a slight distal motor deficit in the left lower limb (4/5 on tibialis anterior according to the Medical Research Council scale) and a spastic gait pattern with greater involvement of the left side. No evidence of lower motor neuron impairment, bulbar involvement, or sensory disturbance was observed.

**Figure 1:**
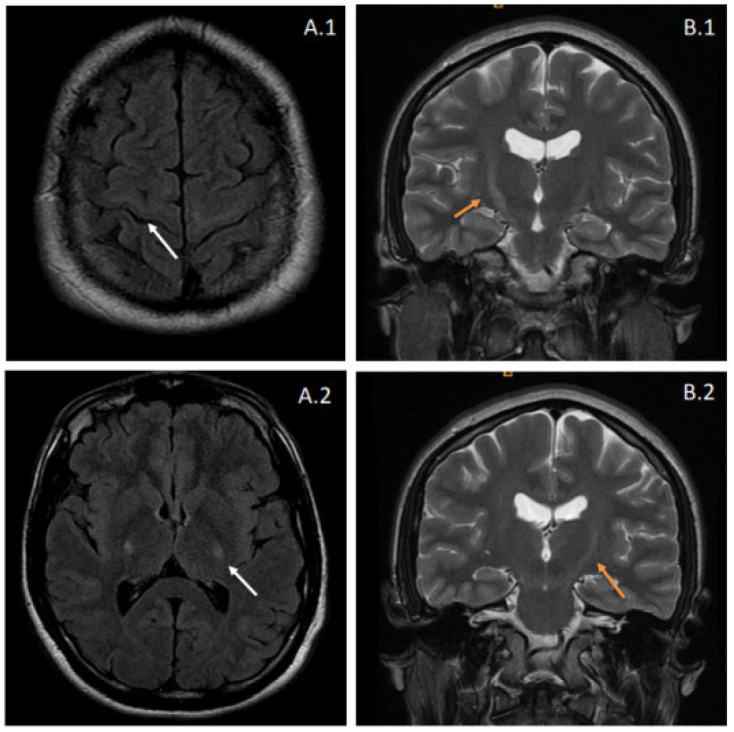
Brain MRI from the proband. Images (**A.1**,**A.2**) correspond to fluid-attenuated inversion recovery (FLAIR) sequences, showing hyperintensity affecting the pyramidal tracts bilaterally (white arrows). These features suggest degeneration of the corticospinal pathway and can also be observed in coronal T2-weighted images (**B.1**,**B.2**) as increased signal intensities at the internal capsule. The latter radiologic appearance is also known as the “Wine Glass” sign.

Brain and spinal magnetic resonance imaging (MRI) showed T2-weighted and fluid-attenuated inversion recovery (FLAIR) increased signal intensities at the pyramidal tracts bilaterally, suggestive of corticospinal pathway degeneration (Figure 1). The electrophysiological tests showed evidence of lower motor neuron involvement, with active denervation in lower extremity muscles in needle electromyography (tibialis anterior, vastus medialis, and gastrocnemius bilaterally) and a conduction defect in the corticospinal pathway in both upper and lower extremities using transcranial magnetic stimulation. Cerebrospinal fluid analysis was entirely normal. Further comprehensive laboratory workup ruled out other inflammatory or infectious causes.

The evolution in the following months showed a fast progression of the disorder. Slight signs of lower motor neuron lesion appeared in the spinal territory with sporadic fasciculations in the upper and lower extremities. The pyramidal syndrome spread to the upper extremities and bulbar region, and the patient developed spastic dysarthria and dysphagia. The distal weakness in the left lower extremity and spasticity progressively worsened, losing independent walking ability eight months after onset. A diagnosis of clinically probable ALS was made according to the revised El Escorial criteria [9] and treatment with riluzole 50 mg twice was initiated.

There was no family history of motor neuron diseases, dementia, or psychiatric disorders. The patient was an only child born of non-consanguineous parents. His father was 61 years old, and his mother was 62 years old at the moment of the evaluation. Both parents were examined as well and were clinically unaffected, and had no health complaints of neurologic nature. Considering the clinical diagnosis and age of onset, genetic studies were performed.

### 2.2. Genetic Results

*SOD1* and *C9orf72* were studied first, given that they are the most prevalent genes associated with ALS in the European population [2] and the emerging therapies for ALS due to pathogenic variants in these genes (NCT04972487, NCT049937557). Sanger sequencing of *SOD1* showed no pathogenic variants. The pathogenic repeat expansion of *C9orf72* was discarded by polymerase chain reaction (PCR) amplification, subsequent analysis of the fragments by capillary electrophoresis, and confirmation of the GGGGCC hexanucleotide expansion by repeat-primed PCR (RP-PCR).

Subsequently, we performed exome sequencing prioritizing variants in genes associated with ALS and frontotemporal dementia (FTD) as well as genes based on appropriate HPO terms. These terms and the analyzed genes are listed as Supplementary Materials. A heterozygous variant c.1035C > G (p.Asn345Lys) was detected in the *TARDBP* gene (NM_007375.3). This variant was considered according to the American College of Medical Genetics (PS1, PS4, PM1, PM2) [10]. It has not been previously described in general population databases (GnomAD), but has been in several patients with this condition [11,12,13]. An additional heterozygous variant c.2961C > G (p.Phe987Leu) in the *NEK1* gene (NM_012224.2) was identified and classified as a variant of uncertain significance.

Segregation analysis in the parents showed that the pathogenic *TARDBP* variant was inherited from the father, whereas the *NEK1* variant was inherited from the mother.

## 3. Discussion

In this case report, we present a patient with genetically determined ALS caused by a pathogenic variant in the *TARDBP* gene associated with a remarkably atypical phenotype mainly for two reasons: early age of onset, compatible with JALS, and the significant predominance of upper motor neuron involvement. The patient carried an additional variant in *NEK1*, classified as of uncertain significance.

**Figure 2:**
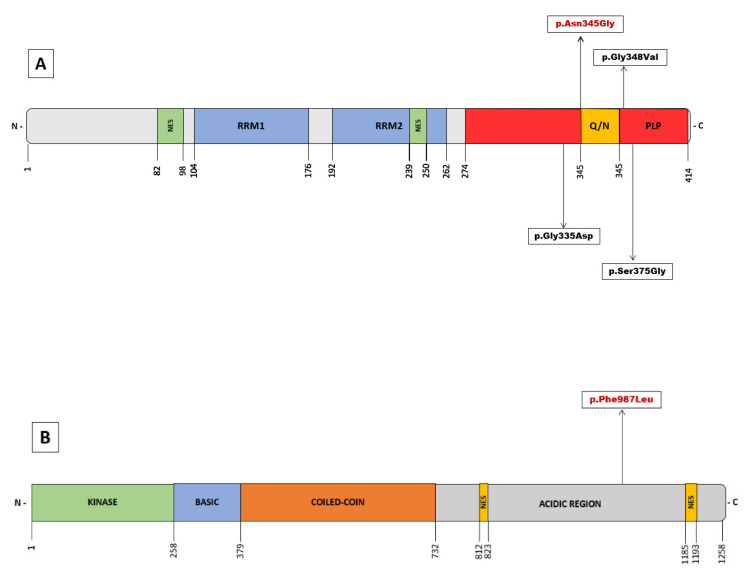
(**A**) Overview of pathogenic variants in *TARDBP* identified in Juvenil ALS. Schematic domain structure of TAR DNA-binding protein 43 (TDP-43). (**B**) Schematic representation of the NEK1 protein structure with the identified variant of uncertain significance. The numbers represent amino acid lengths.

The *TARDBP* gene encodes for the TAR DNA-binding protein-43 (TDP-43), a highly conserved DNA/RNA binding protein with a ubiquitous expression that belongs to the heterogeneous nuclear ribonucleoprotein (hnRNP) family. In 2006, intraneuronal ubiquitinated cytoplasmic inclusions, observed in numerous pathological ALS and FTD specimens, were found to have TDP-43 as a major component [14,15]. These neuronal aggregates of hyperphosphorylated TDP-43 in the brain and spinal cord are considered a histopathological hallmark of the disease and are identified in the vast majority of ALS patients, including sporadic forms and those carrying pathogenic variants (with a few exceptions, such as *SOD1* or *FUS* genes). TDP-43 exerts numerous cellular functions, and multiple mechanisms have been implicated in its pathogenesis [16]. It plays a central role in RNA metabolism, regulating transcription, translation, splicing, mRNA stability and transport, and microRNA maturation. It is also relevant in neuronal defense mechanisms against oxidative stress, and the proper functioning of mitochondrial respiratory chain pathways. Reflecting these functions, mitochondrial localization motifs (M1-M3) and RNA recognition motifs (RRM1 and RRM2) are found in their 414 amino acid structure (Figure 2). In addition, there are sequences of the nuclear localization signal (NLS) and nuclear export signal (NES) because, in physiological conditions, TDP-43 translocates between the nucleus and the cytoplasm to perform these functions [17]. In 2008, mutations in *TARDBP* were first described as a dominant cause of genetic ALS [18,19,20], and to date, more than 48 pathogenic variants have been identified [2]. The majority of these variants are missense and located in the C-terminal region. A low-complexity domain with glycine-rich and glutamine/asparagine-rich (Q/N) sequences is situated here and is considered a prion-like protein (PLP) domain [16].

The variant identified in our patient had only been described in patients with an adult-onset disease [11,12,13], comprising a total of 4 patients from 3 different families, all of them of Asian origin and with symptom onset after 60 years. A variant at the same position but with a different amino acid substitution (c.1035C > A) is a well-known pathogenic variant in adult-onset ALS, included in most genetic databases and used in various molecular models of the disorder [21,22]. *TARDBP*-associated ALS cases develop a classic ALS phenotype with a mean age of onset of 53 years but with wide variability in both onset and duration [23,24]. The most distinctive phenotypic feature is a more frequent onset in the upper extremities, but with a similar evolution to the sporadic ALS, including the extension to other regions and bulbar involvement. To our knowledge, only four cases of JALS associated with three different variants in the *TARDBP* gene have been described [25,26,27,28] (Table 1). An overview of the clinical data of these cases reveals the following. In three cases, the clinical onset was in the upper limbs. In all cases, the initial motor symptoms and those causing the most disability corresponded to predominantly lower motor neuron symptoms, such as weakness and amyotrophy. Disease duration ranged from 24 to 48 months, although one patient remained alive at the publication date (more than 120 months from onset). In common with most adult-onset pathogenic variants, they are dominant missense variants and are located in the C-terminal region *(*Figure 2A). Two of these pathogenic variants associated with *TARDBP-JALS* have also been reported in adult-onset individuals [29,30,31,32,33,34,35]. Despite the limitation of the small number of cases and the absence of detailed clinical descriptions, a comparison of the phenotypes does not reveal a major preference for upper motor neuron involvement as seen in our patient.

In the present case, the variant was inherited from the father’s proband, which shows no clinical or neurophysiological evidence of motor neuron disease or cognitive impairment. Although other members of the paternal family could not be studied, a complete family pedigree history was obtained with no significant findings. It raises the possibility of incomplete or age-dependent penetrance, as has been previously described in the literature. The main series of patients with *TARDBP* pathogenic variants show a median age at disease onset of 53.5 years +/− 12.3 SD [23]. Although there is no detailed information on the overall penetrance of the pathogenic variants in this gene, there are reports of incomplete penetrance in FTD-*TARDBP* [36] and ALS. The most comprehensive research on the risk of ALS in patients carrying a pathogenic variant in *TARDBP* has been carried out in the Sardinian population, given the high prevalence of the p.(Ala382Thr) pathogenic variant. Several studies in this population revealed a penetrance approximately of 60% at 70 years of age but with differences between sexes, pointing to a higher penetrance in men (74–80%) than in women (42.5–66%) [37].

In addition to the pathogenic variant in *TARDBP*, the proband presented an additional variant in the *NEK1* gene, p.(Phe987Leu), which is classified as a variant of unknown significance, and its role in the pathogenesis of our patient is uncertain. *NEK1* was recently described as a gene associated with ALS [38,39] with both loss of function and missense variants postulated as risk factors. *NEK1* encodes for a serine/threonine protein-kinase with functions in cell cycle control, DNA damage repair, cilia regulation, and apoptosis. Loss of function variants in *NEK1* have been associated with an elevated risk of developing ALS [40,41,42]. This variant is located in the acidic region proximal to the C-terminal region but not in any known specific domain (Figure 2B). The distribution of the known pathogenic or likely pathogenic variants in the *NEK1* gene does not show clustering in hot spots but appears to be distributed throughout its structure [43]. This role as a risk factor suggests a synergistic effect with other players, including other ALS-associated genes, thus supporting the hypothesis of oligogenic inheritance in ALS. Some studies have identified a high frequency of *NEK1* variants and other ALS-associated genes in the same patient [43]. Although the pathogenic role of missense variants remains more controversial, recent studies provide evidence for a possible association of ALS with missense variants in *NEK1* gene [43,44]. For example, a patient carrying a pathogenic variant in the *TARDBP* gene also presenting the missense variant p.(Arg261His) in *NEK1*, considered as a phenotype modifier with earlier disease onset, has been described [41]. To our knowledge, beyond the enrichment of pathogenic variants in ALS-associated genes in carriers of *NEK1* variants, other evidence of a functional interaction between both genes has not been described in the literature. In our case, segregation studies showed that the pathogenic *TARDBP* variant was inherited from the father, whereas the rare variant in *NEK1* was inherited from the mother. This finding is consistent with a previous hypothesis suggesting a second-hit model in which *NEK1* variants act as disease modifiers and suggests the possibility of an epistatic effect of both genes in this particular case. This hypothesis could explain the peculiarities of the phenotype, penetrance, intrafamilial variability, and especially the age of onset seen in our patient. However, this statement should be considered a preliminary hypothesis that requires further study.

## 4. Conclusions

This report highlights the heterogeneity of the phenotypic presentation of ALS associated with diverse pathogenic genetic variants. Furthermore, this case provides further evidence that pathogenic variants in the *TARDBP* gene may be an infrequent cause of JALS.

The view of ALS as a disease with multistep pathophysiology emphasizes the accumulation of various hits in different pathological pathways. In this sense, a polygenic inheritance could explain part of the disease variability, and the expansion of new genetic techniques in the coming years will offer new insights into this field.

## Figures and Tables

**Table 1:** Clinical characteristics of Juvenile ALS *TARDBP*-associated patients. FALS: familial ALS. UL: upper limbs. LL: lower limbs. LMN: lower motor neuron. UMN: upper motor neuron. CI: cognitive impairment.

Case No.	Nucleotide Change	Amino Acid Change	Exon	FALS	Gender	Age of Onset (Years)	Region of Onset	Spread to Bulbar Region	Phenotype: Motor Neuron Predominance		CI/ Dementia	Disease Duration (Months)	Major Source of Disability	Reference
									Onset	Last Follow-Up				
1	c.1004G > A	p.Gly335Asp	6	No	Male	20	Spinal(UL)	No	LMN	LMN	No	24	LMN	Corrado et al. [25]
2	c.1043G > T	p.Gly348Val	6	Yes	Male	24	Spinal (UL)	Yes	LMN	Both	No	46	LMN	Liu et al. [26]
3	c.1123A > G	p.Ser375Gly	6	No	Female	22	Spinal (LL)	Yes	LMN	Both	No	48	LMN	Newell et al. [27]
4	c.1043G > T	p.Gly348Val	6	No	Male	24	Spinal(UL)	Yes	LMN	LMN	No	>120 (Alive at the time of publication)	LMN	Wang et al. [28]
5	c.1035C > G	p.Asn345Lys	6	No	Male	24	Spinal(LL)	Yes	UMN	UMN>>LMN	No	Alive at the time of writing	UMN	This study

## Supplementary Materials

The following supporting information can be downloaded at: https://www.mdpi.com/article/10.3390/genes13081483/s1, Human Phenotype Ontology (HPO) terms used and list of genes analysed in blood DNA next-generation exome sequencing study.
