# Supplementary material for: An Atypical Presentation of Upper Motor Neuron Predominant Juvenile Amyotrophic Lateral Sclerosis Associated with TARDBP Gene: A Case Report and Review of the Literature
Source: Genes (Basel). 2022 Aug 19;13(8):1483. doi: 10.3390/genes13081483 (PMC9407925; doi:10.3390/genes13081483)
Supplement: Supplementary file 1 [file genes-13-01483-s001.zip › genes-1854757-supplementary.pdf]

Supplementary file.

1. Human Phenotype Ontology (HPO) terms used.

HP:0007354 (Amyotrophic lateral sclerosis), HP:0002493 (Upper motor neuron dysfunction), HP0002366 (Abnormal motor neuron morphology).

2. List of genes analysed in blood DNA next-generation exome sequencing study.

AAAS, AARS, AARS2, ABCA1, ABCA7, ABCB7, ABCC6, ABCC8, ABCD1, ABHD12, ACADS, ACAT1, ACD, ACER3, ACOX1, ACP2, ACP5, ACSL4, ACTA1, ACTL6B, ADA2, ADAM22, ADAR, ADARB1, ADAT3, ADCY5, ADD3, ADGRG1, ADNP, ADPRHL2, ADSL, AFG3L2, AGA, AGTPBP1, AGTR2, AIFM1, AIMP1, AIPL1, AKT1, ALAD, ALDH18A1, ALDH3A2, ALG11, ALG13, ALG3, ALS2, AMACR, AMPD2, ANG, ANGPTL6, ANK3, ANKLE2, ANOS1, ANXA11, AP1S2, AP3B2, AP4B1, AP4E1, AP4M1, AP4S1, AP5Z1, APC, APC2, APOA1, APOPT1, APP, ARF1, ARFGEF2, ARG1, ARHGAP31, ARHGEF6, ARHGEF9, ARL13B, ARL6IP1, ARNT2, ARSA, ARSI, ARV1, ARX, ASAH1, ASH1L, ASNS, ASPA, ASXL3, ATAD1, ATAD3A, ATL1, ATM, ATN1, ATP13A2, ATP1A2, ATP1A3, ATP2B3, ATP6AP2, ATP6V0A2, ATP6V1A, ATP6V1E1, ATP7A, ATRX, ATXN1, ATXN10, ATXN2, ATXN3, ATXN7, ATXN8OS, AUH, AUTS2, B3GALNT2, B3GALT6, B4GALNT1, B4GAT1, BAP1, BAZ1B, BCAP31, BCAT2, BCKDHA, BCKDHB, BCL11B, BCOR, BCS1L, BDNF, BEAN1, BICD2, BMPR1A, BOLA3, BRAF, BRAT1, BRF1, BRSK2, BSCL2, BTD, C12orf4, C12orf65, C19orf12, C19orf70, C4A, C9orf72, CACNA1A, CACNA1B, CACNA1D, CACNA1E, CACNA1G, CACNA1S, CACNG2, CAMK2A, CAMK2B, CAMTA1, CAPN1, CARS, CARS2, CASK, CAV1, CBS, CC2D1A, CCDC141, CCDC88C, CCNF, CCR1, CCT5, CDH15, CDK19, CDKL5, CDON, CEP290, CFH, CFHR1, CFHR3, CHAMP1, CHCHD10, CHD7, CHMP1A, CHMP2B, CHP1, CIC, CIT, CKAP2L, CLCF1, CLCN4, CLIC2, CLIP1, CLIP2, CLP1, CLPB, CLTC, CNKSR2, CNOT1, CNP, CNPY3, CNTNAP1, CNTNAP2, COASY, COG2, COG4, COL3A1, COL4A1, COL4A2, COLGALT1, COPB2, COQ2, COQ5, COQ8A, COQ9, COX15, COX20, COX8A, CPT1A, CPT1C, CRADD, CRB1, CRBN, CRLF1, CRX, CSF1R, CSNK2B, CTC1, CTDP1, CTLA4, CTNNA2, CTNNB1, CTSD, CTSF, CUX1, CWF19L1, CXorf56, CYB5A, CYB5R3, CYFIP2, CYP26C1, CYP27A1, CYP2U1, CYP7B1, DALRD3, DAO, DARS, DARS2, DBT, DCC, DCPS, DCTN1, DCX, DDB2, DDC, DDHD1, DDHD2, DDX3X, DEAF1, DEGS1, DENND5A, DHCR24, DHCR7, DHDDS, DHPS, DISP1, DKC1, DKK1, DLAT, DLD, DLG3, DLL1, DLL4, DMD, DMXL2, DNAJC12, DNAJC19, DNAJC3, DNAJC6, DNASE1L3, DNM1, DNM1L, DNMT1, DOCK6, DOCK8, DPAGT1, DPM3, DPYD, DPYS, DSTYK, DUSP6, DYNC1H1, DYNC1I2, DYRK1A, EARS2, EBP, ECHS1, EDC3, EDNRB, EED, EEF1A2, EEF2, EIF2AK2, EIF2AK3, EIF2B1, EIF2B2, EIF2B3, EIF2B4, EIF2B5, EIF2S3, ELN, ELOVL4, ELP2, EMC1, EML1, ENG, ENPP1, ENTPD1, EOGT, EPB41L1, EPCAM, EPHA4, EPM2A, EPRS, ERAP1, ERBB4, ERCC1, ERCC2, ERCC3, ERCC4, ERCC5, ERCC6, ERCC8, ERF, ERGIC1, ERLIN1, ERLIN2, ETHE1, EXOC8, EXOSC3, EXOSC8, EXOSC9, EXT1, EXT2, EXTL3, EYA1, EZH2, EZR, FA2H, FAM126A, FAN1, FAR1, FARS2, FAS, FBLN1, FBXO31, FBXO7, FBXW11, FDX2, FDXR, FEZF1, FGF12, FGF17, FGF8, FGFR1, FIG4, FKRP, FKTN, FLNA, FLRT1, FLRT3, FMN2, FOXG1, FOXH1, FOXP1, FOXRED1, FRMD4A, FRMPD4, FRRS1L, FTL, FTO, FTSJ1, FUCA1, FUS, FXN, GABBR2, GABRA2, GABRA3, GABRA5, GABRB2, GABRD, GABRG2, GAD1, GALC, GAMT, GAN, GAS1, GATAD2B, GBA, GBA2, GBE1, GCDH, GCH1, GDAP2, GDF3, GDF6, GDI1, GFAP, GFM1, GFM2, GJA1, GJB1, GJB2, GJB6, GJC2, GLB1, GLE1, GLI2, GLRA1, GLRB, GLRX5, GLT8D1, GLYCTK, GM2A, GMPPB, GNAO1, GNAQ, GNB1, GOT2, GPAA1, GPHN, GPT2, GRIA2, GRIA3, GRIA4, GRID2, GRIK2, GRIN1, GRIN2A, GRIN2B, GRIN2D, GRM1, GRM7, GRN, GSS, GSX2, GTF2E2, GTF2H5, GTF2I, GTF2IRD1, GTPBP2, GUCY1A1, GUCY2D, GUF1, HACE1, HADHA, HADHB, HCFC1, HCN1, HDAC8, HEPACAM, HESX1, HEXA, HEXB, HIKESHI, HINT1, HIST1H1E, HIVEP2, HK1, HLA-B, HLA-DPA1, HLA-DPB1, HLA-DQB1, HLA-DRB1, HLCS, HMGCL, HNMT, HNRNPA1, HNRNPA2B1, HNRNPH2, HPDL, HPRT1, HS6ST1, HSD17B10, HSD17B4, HSPD1, HSPG2, HTRA1, HTRA2, HTT, HUWE1, IARS, IBA57, IDUA, IFIH1, IFT140, IKBKG, IL10, IL12A, IL17RD, IL1RAPL1, IL23R, IMPDH1, INPP5K, INTS8, IQCB1, IQSEC1, IQSEC2, IRAK1, IREB2, IRF2BPL, ISCA1, ISCA2, ITM2B, JAM2, JAM3, KANK1, KAT6A, KATNB1, KCNA1, KCNA2, KCNA4, KCNAB2, KCNB1, KCNC3, KCNJ13, KCNJ18, KCNJ6, KCNQ2, KCNQ3, KCNQ5, KCNT1, KDM1A, KDM5B, KDM5C, KIDINS220, KIF11, KIF1A, KIF1C, KIF2A, KIF5A, KIF5C, KIRREL3, KISS1R, KLC2, KLRC4, KMT2A, KMT2B, KRAS, KY, KYNU, L1CAM, L2HGDH, LAGE3, LAMB1, LARGE1, LBR, LCA5, LIAS, LIMK1, LIMS2, LINGO1, LINS1, LIPT1, LIPT2, LMAN2L, LMNB1, LMX1B, LRAT, LRP4, LRRK2, LYRM7, LYST, MACF1, MAG, MAN1B1, MAN2B1, MAPK8IP3, MAPT, MARS, MARS2, MATR3, MBD5,

MBOAT7, MC2R, MCCC1, MCCC2, MCOLN1, MDH1, MDH2, MECP2, MECR, MED12, MED12L, MED13L, MED17, MED23, MED25, MEFV, MEOX1, METTL23, METTL5, MFF, MFN2, MFSD2A, MGAT2, MID2, MIPEP, MLC1, MLH1, MLH3, MOCS1, MOCS2, MORC2, MPDU1, MPLKIP, MRAP, MRE11, MRM2, MRPS22, MRPS34, MSH2, MSH6, MSL3, MTFMT, MTHFR, MTHFS, MT-ND1, MTO1, MTOR, MTPAP, MTRR, MTTP, MUT, MYO5A, MYORG, MYT1L, NAA10, NACC1, NADK2, NAGA, NAGS, NALCN, NARS, NARS2, NAXE, NCAPD3, NCAPG2, NDE1, NDNF, NDP, NDST1, NDUFA10, NDUFA12, NDUFA13, NDUFA2, NDUFA4, NDUFA6, NDUFA9, NDUFAF2, NDUFAF3, NDUFAF4, NDUFAF5, NDUFAF6, NDUFB8, NDUFS1, NDUFS2, NDUFS3, NDUFS4, NDUFS7, NDUFS8, NDUFV1, NDUFV2, NECAP1, NEFH, NEFL, NEK1, NEU1, NEUROD2, NEXMIF, NF1, NF2, NFASC, NHLRC1, NHLRC2, NIPA1, NIPBL, NKX6-2, NMNAT1, NNT, NODAL, NOP56, NOTCH1, NOTCH3, NOVA2, NPC1, NPC2, NPHP3, NR2F1, NSD1, NSMF, NSUN2, NT5C2, NTNG1, NTNG2, NTRK2, NUBPL, NUP107, NUP133, NUP214, NUP62, NUS1, OCLN, ODC1, OGDH, OPA1, OPA3, OPHN1, OPTN, OSGEP, OSTM1, OTUD6B, OTX2, PAFAH1B1, PAH, PAK3, PANK2, PARK7, PARN, PARS2, PAX1, PAX3, PAX6, PCBD1, PCCA, PCCB, PCDH12, PCLO, PCYT1A, PCYT2, PDCD1, PDGFB, PDGFRB, PDHA1, PDHX, PDYN, PET100, PET117, PEX1, PEX10, PEX11B, PEX12, PEX13, PEX14, PEX16, PEX19, PEX2, PEX26, PEX3, PEX5, PEX6, PEX7, PFN1, PGAP1, PHACTR1, PHGDH, PHYH, PI4KA, PIGA, PIGC, PIGN, PIGP, PIGQ, PIGT, PIGU, PIK3CA, PIK3R5, PINK1, PITX3, PLA2G6, PLAA, PLCB1, PLEKHG5, PLK4, PLP1, PLPBP, PMPCA, PMPCB, PMS1, PMS2, PNKP, PNP, PNPLA6, PNPLA8, PNPO, PNPT1, PODXL, POLA1, POLG, POLG2, POLR1C, POLR3A, POLR3B, POMGNT1, POMK, POMT1, POMT2, PON1, PON2, PON3, POU3F3, PPARGC1A, PPOX, PPP1R15B, PPP2R2B, PPP3CA, PPT1, PQBP1, PRDM16, PRDM8, PRF1, PRICKLE1, PRKN, PRKRA, PRNP, PROK2, PROKR2, PRPH, PRPS1, PRRT2, PRSS12, PRTN3, PRUNE1, PSAP, PSAT1, PSEN1, PSEN2, PSPH, PTCH1, PTCHD1, PTEN, PTPN22, PTPN23, PTS, PUM1, PYCR2, QDPR, RAB11A, RAB11B, RAB18, RAB27A, RAB39B, RAB3GAP1, RAB3GAP2, RAD21, RAD50, RALGAPA1, RANBP2, RARS, RARS2, RASA1, RBM28, RBPJ, RD3, RDH12, REEP1, REEP2, REPS1, RERE, RETREG1, RFC1, RFC2, RFT1, RHOBTB2, RLIM, RNASEH1, RNASEH2A, RNASEH2B, RNASEH2C, RNASET2, RNF113A, RNF13, RNF170, RNF216, ROGDI, RORA, RPE65, RPGRIP1, RPIA, RPS20, RPS6KA3, RRM2B, RSRC1, RTEL1, RTN2, RTTN, RUSC2, SACS, SAMD9, SAMD9L, SAMHD1, SARDH, SARS, SATB2, SCN1A, SCN1B, SCN2A, SCN3A, SCN4A, SCN8A, SCO2, SCYL1, SCYL2, SDHA, SDHAF1, SDHB, SDHD, SEC31A, SELENOI, SEMA3A, SEMA4A, SEPSECS, SERAC1, SET, SETBP1, SETD5, SETX, SHH, SHMT2, SIGMAR1, SIK1, SIL1, SIX3, SIX6, SKI, SLC12A2, SLC12A5, SLC12A6, SLC13A5, SLC16A2, SLC17A5, SLC18A2, SLC19A3, SLC1A2, SLC1A3, SLC1A4, SLC20A2, SLC25A10, SLC25A12, SLC25A15, SLC25A19, SLC25A22, SLC25A4, SLC25A46, SLC2A1, SLC2A3, SLC30A10, SLC30A9, SLC33A1, SLC35A2, SLC35C1, SLC39A14, SLC44A1, SLC45A1, SLC52A2, SLC5A6, SLC6A19, SLC6A3, SLC6A5, SLC6A8, SLC6A9, SLC9A7, SMARCB1, SMARCE1, SMC1A, SMC3, SMO, SMPD1, SMPD4, SNCA, SNX14, SOD1, SON, SORL1, SOX10, SOX2, SOX3, SOX4, SP110, SPART, SPAST, SPATA5, SPATA7, SPG11, SPG21, SPG7, SPOP, SPP1, SPR, SPRY4, SPTAN1, SPTBN2, SQSTM1, SRPX2, ST3GAL3, STAG2, STAMBP, STAR, STAT4, STN1, STUB1, STXBP1, SUCLA2, SUFU, SUMF1, SUOX, SURF1, SUZ12, SVBP, SYNE1, SYNGAP1, SYNJ1, SYP, SZT2, TACO1, TACR3, TAF1, TAF15, TAF2, TANGO2, TAOK1, TARDBP, TARS, TARS2, TBC1D20, TBC1D23, TBC1D24, TBCD, TBCE, TBK1, TBL1XR1, TBL2, TBP, TCF20, TCF4, TCTN2, TDGF1, TDP1, TECPR2, TECR, TELO2, TERT, TFG, TGFB1, TGFBR2, TGFBR3, TGIF1, TGM6, TH, THG1L, THOC2, THSD1, TIMM50, TIMM8A, TINF2, TLR3, TLR4, TMEM106B, TMEM231, TMEM63A, TMEM67, TMTC3, TMX2, TNFRSF11A, TNIK, TOE1, TOMM40, TOR1A, TP53, TP53RK, TPI1, TPK1, TPP1, TPP2, TPRKB, TRAF7, TRAK1, TRAPPC12, TRAPPC2L, TRAPPC4, TRAPPC9, TREM2, TREX1, TRIM8, TRIT1, TRMT10A, TRMT5, TRPM3, TRPM7, TSC1, TSC2, TSEN15, TSEN2, TSEN34, TSEN54, TSPAN7, TTBK2, TTC19, TTPA, TTR, TUBA1A, TUBA4A, TUBB2B, TUBB3, TUBB4A, TUBG1, TUBGCP2, TUBGCP4, TUBGCP6, TUFM, TULP1, TUSC3, TWNK, TXN2, TXNRD2, TYROBP, UBA5, UBAC2, UBAP1, UBQLN2, UBTF, UCHL1, UFC1, UFM1, UGDH, UGP2, UNC13A, UNC80, UPF3B, USP27X, USP45, USP8, USP9X, VAC14, VAMP1, VAPB, VCP, VPS11, VPS13A, VPS13C, VPS13D, VPS37A, VPS53, VWA3B, WARS2, WASHC4, WASHC5, WDR11, WDR26, WDR4, WDR45, WDR45B, WDR48, WDR62, WDR73, WT1, WWOX, XPA, XPC, YIF1B, YWHAG, ZC3H14, ZC4H2, ZEB2, ZFR, ZFYVE26, ZFYVE27, ZIC2, ZMYND11, ZNF335, ZNF41, ZNF592, ZNF711, ZNF81, ZSWIM6.
